# Exploring the Need for a Consensus Guideline for the Management of Non-Muscle-Invasive Bladder Cancer: A Scoping Review

**DOI:** 10.7759/cureus.93553

**Published:** 2025-09-30

**Authors:** Obinna Enemoh, Mayowa Adefehinti, Quadri A Sanni, Hykmat A Ogunbadejo, Daniel Brabi, Obichukwu Iwunna, Stephen O Agboro, Bernard Chukwumah, Abiodun Akintayo, Henry I Njeakor, Gentle C Uwaoma

**Affiliations:** 1 General Surgery, Chesterfield Royal Hospital, Chesterfield, GBR; 2 Urology, Peterborough City Hospital, Peterborough, GBR; 3 Urology, South Warwickshire University NHS Foundation Trust, West Midlands, GBR; 4 Surgery, Lagos University Teaching Hospital, Lagos, NGA; 5 Urology, College of Medicine, University of Lagos, Lagos, NGA; 6 Surgery, University of Health and Allied Sciences, Ho, GHA; 7 Surgery, Surgery Interest Group of Africa, Lagos, NGA; 8 General Practice, NHS Health Education England East Midlands, Mansfield, GBR; 9 Emergency Medicine, Mid Cheshire Hospitals NHS Foundation Trust, Crewe, GBR; 10 General Surgery, University Hospital Lewisham, London, GBR; 11 General Practice, New Cross Hospital, Wolverhampton, GBR; 12 Surgery, University Hospital Southampton NHS Foundation Trust, Southampton, GBR; 13 Urology, Hafar al-Batin Central Hospital, Hafar al-Batin, SAU; 14 Internal Medicine, College of Medicine, University of Nigeria, Enugu, NGA

**Keywords:** bladder cancer, cancer chemotherapy, muscle-invasive bladder cancer, nmibc, nmibc treatment, non-muscle-invasive bladder cancer, onco-urology

## Abstract

Non-muscle-invasive bladder cancers (NMIBC) are a heterogeneous subclass of bladder cancers consisting of carcinoma in situ, stage Ta disease, and stage T1 disease. Despite treatment by tumor resection, they have a high rate of recurrence and progression, which present unique management challenges. This scoping review discusses the management of NMIBC, including risk stratification, intravesical therapy, surveillance protocols, and developing treatments. A systematic search in different databases (PubMed/MEDLINE, Embase, Cochrane Library, and Web of Science) was carried out according to the Preferred Reporting Items for Systematic Reviews and Meta-Analyses extension for Scoping Reviews (PRISMA-ScR) guidelines. Publications in English, from January 2010 to April 2025, were included. The final search was conducted on May 5, 2025. A total of 16 studies that met our inclusion criteria were reviewed.

Different themes emerged, which include risk stratification and diagnosis, tumor resection, intravesical therapy, surveillance and follow-up, patient factors, and novel therapies. Substantial differences were found in clinical practice. Immediate postoperative chemotherapy was not adequately used, and the schedules of surveillance varied. Newer therapies, such as immune checkpoint inhibitors and novel intravesical agents, are promising. The role of enhanced cystoscopy and urinary biomarkers is also increasing for non-invasive disease monitoring. Despite this advancement, therapy standardization or a patient-centered view is still lacking. This review highlights the need for harmonized guidelines, wider access to innovative therapies, and collaborative research to improve outcomes in patients living with NMIBC.

## Introduction and background

Bladder cancer is the 10th most common malignancy in the world, with 573,000 new cases and over 200,000 deaths projected to have occurred worldwide in 2020 [1]. Non-muscle-invasive bladder cancer (NMIBC) is a heterogeneous subclassification of bladder cancer consisting of carcinoma in situ, stage Ta disease, and stage T1 disease [2]. NMIBC represents approximately 75% of newly diagnosed bladder cancer subtypes. It has a better prognosis compared to muscle-invasive disease. Its clinical burden is difficult because of high recurrence rates and the risk of progressing to muscle-invasive forms [3]. There is a need for a balance between reducing recurrence and progression, minimizing the burden and adverse effects of treatment, and making its management complex. 

Standard initial management is transurethral resection of bladder tumor (TURBT) with risk-adapted intravesical therapies like Bacillus Calmette-Guérin (BCG), immunotherapy, or intravesical chemotherapeutic agents such as mitomycin C [4]. The European Association of Urology (EAU), American Urological Association (AUA), and National Institute for Health and Care Excellence (NICE) have different guidelines for the grouping and management of different risk groups. Long-term surveillance with cystoscopy and urine cytology is essential, increasing the clinical and economic burden on the patient [5]. Despite existing guidelines, their clinical applications vary. This is because of different patient characteristics, availability of healthcare resources, clinician preference of treatment options, and evolving evidence-based practice. Emerging therapies like immune checkpoint inhibitors, device-assisted therapies like chemohyperthermia, and newer forms of intravesical agents make management choices complex. As a result, the management of NMBIC needs continuous review to guide both practice and research.

This scoping review aims to examine current literature in the management of NMIBC. It accesses available options for treatment and surveillance in NMIBC and common themes and differences in management protocols among different healthcare systems and guidelines. Objectives are to highlight areas of current research shortcomings, long-term outcomes, treatment tolerability, and patient perspectives. This will present an overview of the current state of knowledge, influence the direction of future research, and help clinicians and policymakers to improve the strategies of NMIBC management.

## Review

Aims and objectives

The aims were to define different treatment and surveillance strategies, compare clinical guideline recommendations, and examine areas of knowledge deficiency and research preferences. The objective is to describe the current management of NMIBC in adult patients.

Study design

This scoping review was performed according to the Arksey and O'Malley methodological framework [6]. There was no registration of this review in any repository as per the exemptions for scoping reviews. Reporting was guided by the Preferred Reporting Items for Systematic Reviews and Meta-Analyses extension for Scoping Reviews (PRISMA-ScR) checklist [7].

Ethical considerations

Ethical approval was waived since it only included published, publicly available literature and did not involve any human participants.

Eligibility criteria

The Population-Concept-Context (PCC) framework was used for eligibility purposes [8]. Adults diagnosed with NMIBC were the study population. The concept describes management strategies, including surgical options and intravesical therapies, while the context involves all countries and healthcare systems. Inclusion criteria were peer-reviewed research, including clinical trials, cohort studies, reviews, and guideline documents. Publications in English, from January 1, 2010, to April 30, 2025, were searched to show recent management approaches. The final search was conducted on May 5, 2025. Exclusion criteria were case reports, editorials, opinion papers, and abstracts from conferences that did not have enough data. Investigations based on animal models or in vitro discoveries were also excluded.

Information sources and search strategy

A literature search across electronic databases listed below has generated records: PubMed (MEDLINE), Embase, Cochrane Library, and Web of Science. Controlled vocabulary for the search was generated with Medical Subject Headings (MeSH) and other keywords (‘Non-Muscle Invasive Bladder Neoplasms’, ‘Bladder Cancer, Non-Muscle-Invasive’, ‘NMIBC’). Boolean operators and filters were used to increase sensitivity.

All records were bound and fed into EndNote X9 (Clarivate, London, United Kingdom) for reference-inclusion management and duplicate removal. Titles and abstracts were then examined independently by two reviewers using Rayyan QCRI (Rayyan Systems Inc., Cambridge, Massachusetts, United States). Any disagreement between the two reviewers was resolved through an arbitration by a third reviewer. 

Data charting process

A standard data-extraction form was prepared in MS Excel (Microsoft Corporation, Redmond, Washington, United States) following the Joanna Briggs Institute (JBI) guidelines. The following particulars were recorded: author(s), year, country of study, study type, population characteristics, type of NMIBC management discussed, key findings with an emphasis on reported outcome recurrence, progression, adverse event, compliance, etc. Data analysis was descriptive and thematic rather than a meta-analysis. The charted data were grouped and presented under the following themes: Initial Surgical Management, Intravesical Therapy, Surveillance and Follow-Up, Risk Stratification and Prognostic Tools, Clinical Guidelines and Practice Patterns, and Emerging Therapies. Results, where applicable, were stratified by risk group (low, intermediate, high) and by geographic region to study variations in management.

Results

**Figure 1:**
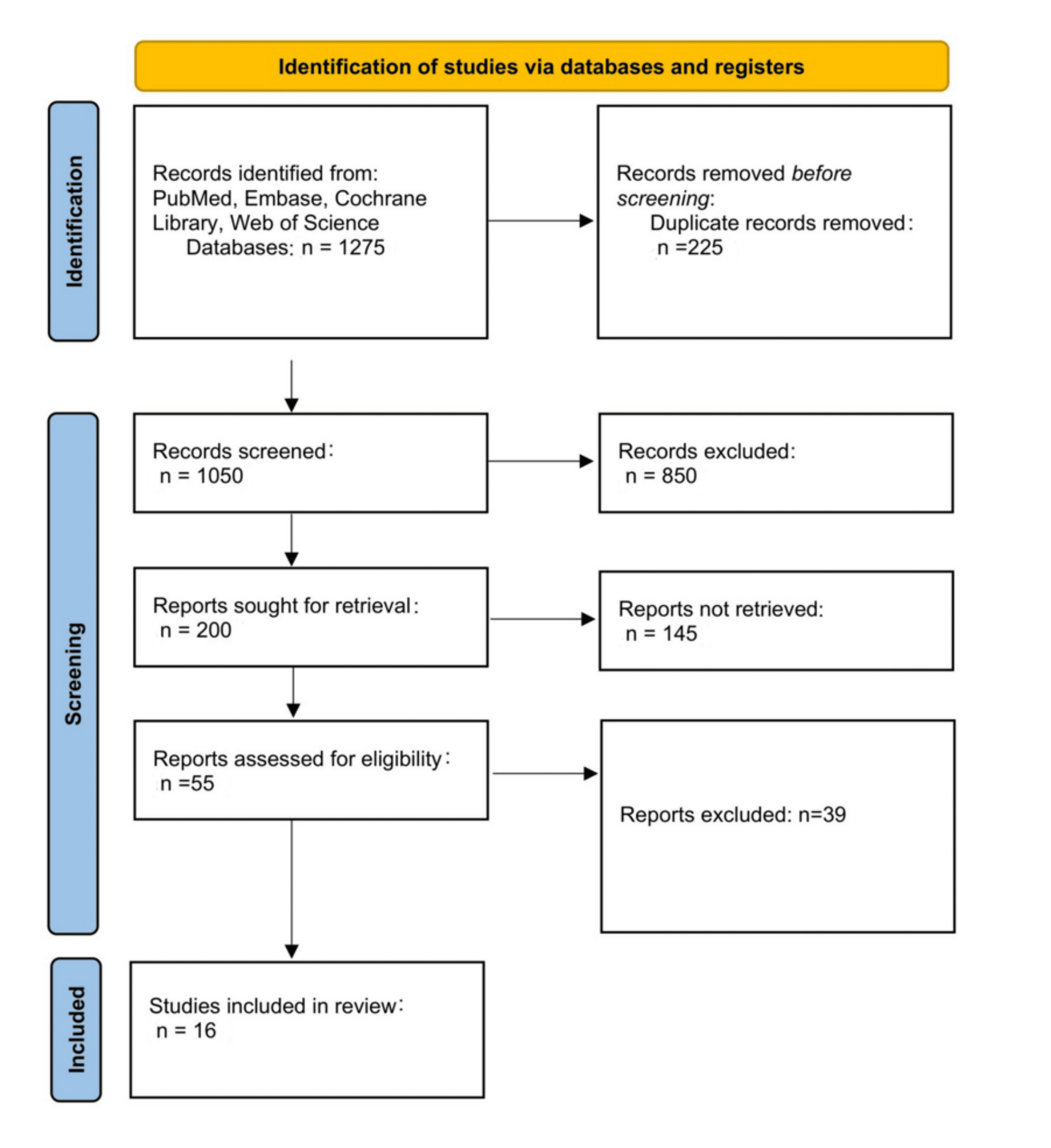
Search strategy PRISMA-ScR: Preferred Reporting Items for Systematic Reviews and Meta-Analyses extension for Scoping Reviews

After a literature search from different databases, including PubMed, Embase, Cochrane Library, and Web of Science, as well as a grey literature manual search, 1,275 articles were identified. Following the removal of duplicates, 1,050 remaining records had their titles screened. Eight hundred and fifty articles did not meet the research question or had insufficient data on management strategy. Two hundred papers underwent an abstract screening followed by full-text retrieval. Abstracts that did not meet all inclusion criteria or had access issues were not retrieved. This resulted in 16 studies included in this synthesis. The PRISMA-ScR flowchart reporting search strategy is illustrated in Figure 1.

Characteristics of the Included Studies

Most of them were from high-income countries: seven from the United States, three from Europe (the United Kingdom and Italy), three from China, two from Canada, and one from Australia. The majority of the studies were systematic reviews (n=7), other literature reviews (n=5), retrospective cohort studies (n=2), clinical trials (n=1), and randomized controlled trials (RCTs) (n=1). The studies included in the review are listed in Table 1. 

**Table 1:** Studies included AUA: American Urological Association; SUO: Society of Urologic Oncology; BCG: Bacillus Calmette-Guérin; RCT: randomized controlled trial

Author	Title	Country of study	Year of publication	Type of study	Population size	Study themes/key findings/datasets from study
Bree et al. [9]	Management, surveillance patterns, and costs associated with low-grade papillary stage Ta non-muscle-invasive bladder cancer among older adults, 2004-2013	USA	2022	Cohort study	13,054	Surveillance, intravesical therapy
Goldberg et al. [10]	Pharmacologic therapies for non-muscle invasive bladder cancer: current and future treatments	USA	2022	Narrative review	N/A	Risk stratification, intravesical therapy, novel therapies
Holzbeierlein et al. [11]	Diagnosis and treatment of non-muscle invasive bladder cancer: AUA/SUO guideline: 2024 amendment	USA	2024	Systematic review	N/A	Diagnosis, risk stratification, surveillance, intravesical therapy
Hurle et al. [12]	Midterm follow-up (3 years) confirms and extends short-term results of intravesical gemcitabine as bladder-preserving treatment for non-muscle-invasive bladder cancer after BCG failure	Italy	2021	Clinical trials	46	Intravesical therapy
Li et al. [13]	Intravesical combination therapies for non-muscle invasive bladder cancer: recent advances and future directions	UK	2022	Systematic review	N/A	Intravesical therapy, novel therapies
Musat et al. [14]	Treatment outcomes of high-risk non-muscle invasive bladder cancer (HR-NMIBC) in real-world evidence (RWE) studies: systematic literature review (SLR)	USA	2022	Systematic review	N/A	Intravesical therapy, surveillance
Shore et al. [15]	Non-muscle-invasive bladder cancer: an overview of potential new treatment options	USA	2021	Systematic review	N/A	Risk stratification, intravesical therapy, novel therapies
Yassaie et al. [16]	Novel and emerging approaches in the management of non-muscle invasive urothelial carcinoma	Canada	2021	Literature review	N/A	Intravesical therapy, novel therapies
Lerner et al. [17]	Failure to achieve a complete response to induction BCG therapy is associated with increased risk of disease worsening and death in patients with high risk non-muscle invasive bladder cancer	USA	2008	RCT	660	Intravesical therapy
Slovacek et al. [18]	Approaches to non-muscle-invasive bladder cancer	USA	2021	Literature review	N/A	Risk stratification, Intravesical therapy, surveillance, novel therapies
Balasubramanian et al. [19]	Adjuvant therapies for non-muscle-invasive bladder cancer: advances during BCG shortage	Australia	2022	Systematic review	N/A	Intravesical therapy, novel therapies
Claps et al. [20]	BCG-unresponsive non-muscle-invasive bladder cancer: current treatment landscape and novel emerging molecular targets	Italy	2023	Literature review	N/A	Intravesical therapy, novel therapies
Jin et al. [21]	Treatment and surveillance for non-muscle-invasive bladder cancer: a clinical practice guideline (2021 edition)	China	2022	Guideline/systematic review	N/A	Risk stratification, intravesical therapy
Chen et al. [22]	The optimal intravesical maintenance chemotherapy scheme for the intermediate-risk group non-muscle-invasive bladder cancer	China	2023	Cohort study	158	Intravesical therapy
Zhang et al. [23]	Management of non-muscle-invasive bladder cancer: quality of clinical practice guidelines and variations in recommendations	China	2019	Systematic review	N/A	Intravesical therapy
Bhindi et al. [24]	Canadian Urological Association guideline on the management of non-muscle-invasive bladder cancer - abridged version	Canada	2021	Non-systematic reviews	N/A	Risk stratification, intravesical therapy

Risk Stratification and Diagnosis

Risk stratification was a common theme in the articles included using the EAU or AUA risk stratification system. Cystoscopy is the mainstay of diagnosis and follow-up. Other radiological investigations in the form of CT urogram or ultrasonography were used widely in diagnosis and workup. The use of urine cytology in diagnosis and surveillance is variable in clinical practice. The newer markers like UroVysion™ and nuclear matrix protein 22 (NMP22) had variable sensitivity, specificity, and practical applications [11,18].

Initial Management: TURBT

All studies recognized TURBT as the main therapeutic approach in NMIBC. High-quality TURBT, with complete tumor resections, was highlighted as a prognostic variable. Re-resection TURBT was indicated in some studies for high-grade Ta or T1 tumors or when the initial resection is incomplete [25]. Recurrence-free survival improved with re-TURBT, and it gave better staging information.

Intravesical Therapy

Post-TURBT, single-dose high-dose intravesical chemotherapy was advocated in many studies. This was favored for use in low- and intermediate-risk patients with a reduction of recurrence risk [25]. BCG therapy was recommended especially for intermediate- and high-risk NMIBC. Induction/maintenance regimens like the Southwest Oncology Group (SWOG) protocol demonstrated better recurrence-free survival for high-risk patients. However, some studies described BCG shortages, adverse effects, and patient intolerance, which were common limitations [19]. Epirubicin, mitomycin C, and gemcitabine were used in induction and maintenance settings if BCG was contraindicated or ineffective. The efficacy was lower than BCG, but the tolerability was better. Alternative regimens using lower doses, different strains, or sequential chemo-immunotherapy were investigated, but evidence has so far been inconclusive.

Surveillance and Follow-Up

Follow-up protocols were specified using EAU and AUA guidelines. Surveillance was by periodic cystoscopy (every six months, 1-2 years, annually thereafter), urine cytology, and upper tract imaging. Higher-risk patients needed a closer follow-up, with concerns over repeat imaging and biopsies. New tools like narrow band imaging (NBI) or blue-light cystoscopy showed an improved detection of recurrent or flat lesions.

Management of Recurrence and Progression

In the management of recurrent disease, response was dependent on risk category and past treatment. Treatment options for BCG failure consisted of BCG re-induction, intravesical chemotherapy alternatives such as gemcitabine-docetaxel combination, device-assisted therapies, electromotive drug administration (EMDA), and hyperthermic intravesical chemotherapy (HIVEC). Early radical cystectomy (RC) was recommended in high-risk, BCG-unresponsive cases. Three studies highlighted the monitoring of the timing of RC and good outcomes when it is done before the development of muscle invasion.

Novel and Investigational Therapies

Emerging therapies such as immune checkpoint inhibitors like atezolizumab, gene therapy (e.g., nadofaragene firadenovec), and vaccine-based strategies have become available. However, they were in early-phase trials and had limited long-term data. Local exposure and systemic toxicity minimization through the use of drug delivery technologies such as hydrogel-based systems and sustained-release formulations were also studied.

Health System and Patient Factors

Barriers to optimal NMIBC management have become a growing concern. Resource availability affects the quality of TURBT and access to BCG. Adherence to guidelines was noted to be variable across institutions and providers. Patient comorbidities and preferences influence treatment choices, limiting the effectiveness of management. Delays in referrals and follow-up are associated with worse outcomes and significantly affect management. Patient-reported outcome measures, such as quality of life or treatment burden, were rarely reported and need to be researched more broadly.

Discussion 

Classifications developed by the EAU and AUA recommend the individualization of treatment. Risk stratification continues to be the cornerstone of NMIBC management [26]. Low-risk patients respond well to conservative strategies, while high-risk patients require more aggressive intervention, maintenance of BCG, or early RC. However, despite the consistent use of risk frameworks, there were differences in implementation based on institutional policies, patient preferences, or regional guideline dissimilarities [9,24].

TURBT is universally accepted as a first step in NMIBC treatment [27]. Re-TURBT may be necessary in high-grade and T1 tumors to ensure complete resection and accurate staging. This review reported strong evidence in favor of high-quality TURBT as a predictor of recurrence and progression. Nevertheless, technical variations, such as surgeon experience or use of enhanced imaging and blue-light cystoscopy, were frequently not standardized, contributing to differences in outcome and being a factor in comparing studies. Single-dose intravesical chemotherapy after TURBT provides strong anticancer active effects in low- to intermediate-risk patients [10]. In spite of these guidelines, several studies reported its underutilization as a result of logistical barriers like drug availability and delays in drug administration post-TURBT. These operational issues represent a gap between evidence-based recommendations and actual practice.

BCG has good outcomes as intravesical therapy for intermediate- and high-risk NMIBC. Its use with maintenance regimens lasting up to three years is supported by consistent studies. However, its application is limited by the availability of BCG and dangers associated with its use such as cystitis and systemic effects [28]. The shortage of BCG all over the world in 2020 led researchers and clinicians to look for alternative protocols, for example, dose reductions or antibodies to other intravesical agents. Despite the reasonable effectiveness of reduced-dose BCG in several trials, its long-term effect on disease progression is not clear. Consensus around the optimal management of BCG-unresponsive NMIBC is still uncertain, with early cystectomy being curative but with high morbidity. Mitomycin C, gemcitabine, and epirubicin are common alternatives to the therapy as an option for BCG-intolerant or BCG-unresponsive patients. Various studies state gemcitabine is as effective as mitomycin C with fewer side effects. Combination regimens like sequential gemcitabine-docetaxel hold promise, particularly in patients unfit for cystectomy [29]. Novel modes of drug delivery, HIVEC, and electromotive drug administration are beginning to gain ground in efforts to improve therapeutic outcomes and, at the same time, reduce toxicity [30]. However, most studies of these modalities are small cohort studies or early-phase trials, suggesting a reason to pursue larger, multicenter RCTs.

Surveillance after treatment is important because it has a high recurrence rate. Most studies followed the recommended cystoscopic follow-up, with greater frequency if high risk. However, patient factors associated with cystoscopy, like discomfort, cost, and its psychological effects, call for more research into non-invasive adjuncts [31]. New imaging methods, such as NBI and blue-light cystoscopy, have the ability to enhance the detection of a flat or small lesion, but availability has been a problem [32-34]. Biomarkers like NMP22 and UroVysion™ exhibit promise, but the variation in sensitivity and specificity decreases their viability as a single effective test [35]. So, while positive adjunctive tools may increase the accuracy of surveillance, they currently cannot reduce the use of cystoscopy.

BCG-unresponsive NMIBC, perpetual high-grade disease, or disease recurrence within six months of BCG induction presents a complex management dilemma. RC is the gold standard so far in those eligible with the best chance of cure [36]. This is associated with a high morbidity; therefore, bladder conservation strategies are attractive, particularly in older or comorbid individuals. Trials that touch new agents such as immune checkpoint inhibitors (atezolizumab, pembrolizumab), gene therapies (nadofaragene firadenovec), and vaccines are growing progressively. Nadofaragene firadenovec has acquired relevant regulatory approval for use in NMIBC, indicating a prospective shift in care for some patients [37]. More studies are needed to check its long-term effectiveness, safety, and availability. 

Some studies focused on differences in the delivery of care. Inconsistent guideline implementation, specialist accessibility issues, comorbidities among patients, and diagnostic or initiation of treatment delay were common themes. Remarkably, patients have also shied away from aggressive management because of a fear of the quality of life that follows treatment, hence the need for shared decision-making and individualized care planning. Patient-reported outcomes in NMIBC studies are also biased by underreporting. Although clinical endpoints of recurrence and progression are important considerations, others, such as treatment burden, urinary function, and well-being, still require equal consideration in guiding the patients to a holistic management. 

Limitations of the Scoping Review

There were limitations identified in this review. Most of the studies were done in high-income settings, which hinders their generalization within resource-limited environments. The different study designs, the realities of patient demographics, and patient routines made the synthesis narrative rather than a meta-analysis. The number of articles not retrieved for study is another limitation. There was no qualitative assessment of the studies reviewed. No quality appraisal was conducted under the methodology of scoping review, so no judgment was made concerning the validity of the individual study.

Implications for Clinical Practice and Research

This review reinforces the notion that NMIBC management is a dynamic and complex process calling for evidence-based, risk-adapted care. Although there are delineated guidelines, in a true-world scenario, practice is usually disparate because of resource limitations, provider preference, or patient-related factors. As new therapies enter the stage of use in clinical practice, routine oversight, access to new agents, and shared decision-making ought to be a priority. Future research should focus on large-scale and high-quality RCTs on newer intravesical agents and delivery systems, long-term outcomes of bladder-sparing therapies, implications of quality of life, and patient-reported outcome measurement and cost-effectiveness research on new technologies and therapy types.

## Conclusions

This scoping review is a summary of the current management of NMIBC. It is a complex disease requiring a personalized risk-based strategy, and the evidence highlights the role of TURBT, risk stratification, and timely intravesical therapy in driving treatment. The integration of emerging technologies, innovative therapeutic modalities, and a patient-centered approach is the current trend in NMIBC management. While there are guidelines that provide standardized pathways for NMIBC management, this review demonstrates wide variations in clinical use of these pathways. Disparities develop in terms of resource limitations, regional practice variations, patient factors, and systemic limitations in the healthcare system. Surveillance strategies are vital, while adjunctive technologies, including improved imaging techniques and urinary biomarkers, have shown promise. Robust external validation and standardization are required to justify their incorporation into routine clinical workflows. This review also identified a deficit in the incorporation of patient-reported outcomes and quality of life measures in studies on NMIBC. Closing this gap is crucial in promoting shared decision-making, in particular, when trying to balance therapeutic effectiveness versus treatment morbidity in elderly or comorbid populations.

## References

[1] Wéber A, Vignat J, Shah R (2024). Global burden of bladder cancer mortality in 2020 and 2040 according to GLOBOCAN estimates. World J Urol.

[2] Babjuk M, Burger M, Capoun O (2022). European Association of Urology guidelines on non-muscle-invasive bladder cancer (Ta, T1, and carcinoma in situ). Eur Urol.

[3] Cambier S, Sylvester RJ, Collette L (2016). EORTC nomograms and risk groups for predicting recurrence, progression, and disease-specific and overall survival in non-muscle-invasive stage Ta-T1 urothelial bladder cancer patients treated with 1-3 years of maintenance Bacillus Calmette-Guérin. Eur Urol.

[4] Action Bladder Cancer UK (2025) (2025). Treatments. https://actionbladdercanceruk.org/treatments/.

[5] Malmström PU (2020). Cystoscopic surveillance of patients with non-muscle-invasive bladder cancer revisited. Scand J Urol.

[6] Levac D, Colquhoun H, O'Brien KK (2010). Scoping studies: advancing the methodology. Implement Sci.

[7] Tricco AC, Lillie E, Zarin W (2018). PRISMA extension for Scoping Reviews (PRISMA-ScR): checklist and explanation. Ann Intern Med.

[8] Delaney Delaney, L. (2022 (2025). Apply PCC. https://guides.library.unisa.edu.au/ScopingReviews/ApplyPCC.

[9] Bree KK, Shan Y, Hensley PJ (2022). Management, surveillance patterns, and costs associated with low-grade papillary stage Ta non-muscle-invasive bladder cancer among older adults, 2004-2013. JAMA Netw Open.

[10] Goldberg IP, Lichtbroun B, Singer EA, Ghodoussipour S (2022). Pharmacologic therapies for non-muscle invasive bladder cancer: current and future treatments. Arch Pharmacol Ther.

[11] Holzbeierlein JM, Bixler BR, Buckley DI (2024). Diagnosis and treatment of non-muscle invasive bladder cancer: AUA/SUO guideline: 2024 amendment. J Urol.

[12] Hurle R, Contieri R, Casale P (2021). Midterm follow-up (3 years) confirms and extends short-term results of intravesical gemcitabine as bladder-preserving treatment for non-muscle-invasive bladder cancer after BCG failure. Urol Oncol.

[13] Li Y, Youssef SF, Buanz AB (2022). Intravesical combination therapies for non-muscle invasive bladder cancer: recent advances and future directions. Eur J Pharmacol.

[14] Musat MG, Kwon CS, Masters E, Sikirica S, Pijush DB, Forsythe A (2022). Treatment outcomes of high-risk non-muscle invasive bladder cancer (HR-NMIBC) in real-world evidence (RWE) studies: systematic literature review (SLR). Clinicoecon Outcomes Res.

[15] Shore ND, Palou Redorta J, Robert G (2021). Non-muscle-invasive bladder cancer: an overview of potential new treatment options. Urol Oncol.

[16] Yassaie O, Chehroudi C, Black PC (2021). Novel and emerging approaches in the management of non-muscle invasive urothelial carcinoma. Ther Adv Med Oncol.

[17] Lerner SP, Tangen CM, Sucharew H, Wood D, Crawford ED (2008). Failure to achieve a complete response to induction BCG therapy is associated with increased risk of disease worsening and death in patients with high risk non-muscle invasive bladder cancer. Urol Oncol.

[18] Slovacek H, Zhuo J, Taylor JM (2021). Approaches to non-muscle-invasive bladder cancer. Curr Oncol Rep.

[19] Balasubramanian A, Gunjur A, Weickhardt A, Papa N, Bolton D, Lawrentschuk N, Perera M (2022). Adjuvant therapies for non-muscle-invasive bladder cancer: advances during BCG shortage. World J Urol.

[20] Claps F, Pavan N, Ongaro L (2023). BCG-unresponsive non-muscle-invasive bladder cancer: current treatment landscape and novel emerging molecular targets. Int J Mol Sci.

[21] Jin YH, Zeng XT, Liu TZ (2022). Treatment and surveillance for non-muscle-invasive bladder cancer: a clinical practice guideline (2021 edition). Mil Med Res.

[22] Chen JX, Huang WT, Zhang QY (2023). The optimal intravesical maintenance chemotherapy scheme for the intermediate-risk group non-muscle-invasive bladder cancer. BMC Cancer.

[23] Zhang J, Wang Y, Weng H (2019). Management of non-muscle-invasive bladder cancer: quality of clinical practice guidelines and variations in recommendations. BMC Cancer.

[24] Bhindi B, Kool R, Kulkarni GS (2021). Canadian Urological Association guideline on the management of non-muscle-invasive bladder cancer - abridged version. Can Urol Assoc J.

[25] Goldberg IP, Lichtbroun B, Singer EA, Ghodoussipour S (2022). Pharmacologic therapies for non-muscle invasive bladder cancer: current and future treatments. Arch Pharmacol Ther.

[26] Soria F, Rosazza M, Livoti S (2024). Clinical validation of the intermediate-risk non-muscle-invasive bladder cancer scoring system and substratification model proposed by the International Bladder Cancer Group: a multicenter young academic urologists urothelial working group collaboration. Eur Urol Oncol.

[27] Aldousari S, Kassouf W (2010). Update on the management of non-muscle invasive bladder cancer. Can Urol Assoc J.

[28] Guallar-Garrido S, Julián E (2020). Bacillus Calmette-Guérin (BCG) therapy for bladder cancer: an update. Immunotargets Ther.

[29] McElree IM, Steinberg RL, Martin AC (2022). Sequential intravesical gemcitabine and docetaxel for Bacillus Calmette-Guérin-naïve high-risk nonmuscle-invasive bladder cancer. J Urol.

[30] Álvarez-Maestro M, Guerrero-Ramos F, Rodríguez-Faba O, Domínguez-Escrig JL, Fernández-Gómez JM (2021). Current treatments for BCG failure in non-muscle invasive bladder cancer (NMIBC). Actas Urol Esp (Engl Ed).

[31] Kukreja JB, Schroeck FR, Lotan Y (2022). Discomfort and relieving factors among patients with bladder cancer undergoing office-based cystoscopy. Urol Oncol.

[32] Hsueh TY, Chiu AW (2016). Narrow band imaging for bladder cancer. Asian J Urol.

[33] Cahill EM, Chua K, Doppalapudi SK, Ghodoussipour S (2022). The use of blue-light cystoscopy in the detection and surveillance of nonmuscle invasive bladder cancer. Curr Urol.

[34] Zang Z, Wu Q, Chiong E (2019). Blue-light cystoscopy and narrow-band imaging in bladder cancer management. Formos J Surg.

[35] Wan X, Wang D, Zhang X, Xu M, Huang Y, Qin W, Chen S (2025). Unleashing the power of urine‑based biomarkers in diagnosis, prognosis and monitoring of bladder cancer (review). Int J Oncol.

[36] Broughton EI, Chun DS, Gooden KM, Mycock KL, Rajkovic I, Taylor-Stokes G (2022). Treatment and disease management patterns for Bacillus Calmette-Guérin unresponsive nonmuscle invasive bladder cancer in North America, Europe and Asia: a real-world data analysis. Curr Urol.

[37] Steinmetz AR, Mokkapati S, McConkey D, Dinney CP (2024). The evolution of nadofaragene firadenovec: a review and the path forward. Bladder Cancer.

